# Comparison of Animation Distraction Versus Local Anesthetic Application for Pain Alleviation in Children Undergoing Intravenous Cannulation: A Randomized Controlled Trial

**DOI:** 10.7759/cureus.43610

**Published:** 2023-08-16

**Authors:** Aparna R Thomas, Deepa T Unnikrishnan, Isac Mathai M.

**Affiliations:** 1 Paediatrics, Malankara Orthodox Syrian Church Medical Mission Hospital, Kolenchery, Ernakulam, IND

**Keywords:** local anesthetic, less pain, pain on vas, audiovisual distraction, intravenous cannulation

## Abstract

Background

Medical procedures induce behavioral discomfort, fear, and worry in children and their families, worsening their agony. Reading, playing video games, and watching television lessen anxiety and discomfort. This study aims to compare the pain reduction in children using animation distraction and two percent lignocaine with the control group undergoing intravenous (IV) cannulation using the Visual Analogue Scale (VAS) at a tertiary care hospital in Kolenchery, Kerala, South India, and to study the clinico-social factors influencing pain reduction in children undergoing IV cannulation.

Materials and methods

This is an open-label, randomized controlled trial study of 60 children admitted in a pediatric ward, Intensive Care Unit (ICU), or emergency department randomly assigned to either two percent lignocaine application, animation distraction, or control during intravenous cannulation. Children aged six to twelve years requiring IV cannulation for different illnesses were included. Twenty children were randomly assigned to the lignocaine group, twenty to the distraction group, and twenty to the control group. The visual analogue scale was used to measure the subjective pain intensity of the children during IV cannulation. We did statistical analysis using SPSS software version 21 (IBM Corp., Armonk, NY).

Results

Age, gender, previous history of cannulation, site, and size of the cannula were not significantly different between the groups. We did not relate the education of the mother to the VAS scores. The mean VAS score for pain at zero, one, and five minutes was lower in the distraction group compared to the lignocaine and control groups. The mean VAS score for pain at zero, one, and five minutes was not superior among the lignocaine group compared to the control group.

Conclusion

Based on the findings, animation distraction is preferable to lignocaine to alleviate pain in children requiring IV cannulation for a variety of disorders. Distraction is one of the nonpharmacological techniques that seek to alleviate pain by encouraging the patient to focus on something other than the current procedure. In addition to reducing pain and anxiety during excruciating invasive interventions, distraction techniques reduce the number of interventions required and allow for the completion of interventions in less time.

## Introduction

In hospitals, children often experience sudden, intense pain from invasive procedures, which can have negative emotional and mental effects [1]. Children and their families experience behavioral discomfort, fear, and worry because of the medical process, which makes their pain even worse. Procedure-related pain is the discomfort experienced by a conscious patient during a diagnostic or therapeutic procedure. The most terrifying events that children report are those involving medical procedures, notably needle insertions, and they result in the non-cooperation of the children and also their parents [2].

Venipuncture is one of the most painful things that kids have to go through, and half of them hurt while getting it done [3]. Venipuncture differs from other needle procedures like immunization because it takes longer and requires additional medical equipment like tourniquets to find the right vein and draw blood, which causes more anxiety in kids [4].

The child's perception of pain is influenced by a number of variables, including the child's socioeconomic status, family environment, history of exposure to painful activities, and experiences with pharmacologic and non-pharmacologic pain suppression and alleviation [5].

Compared to older children, younger children report more pain with the same stimulus, as well as more anxiety and phobias [6]. Most parents agree and prefer that, besides or instead of medication, non-drug alternatives can help ease the child’s discomfort, make the situation more manageable, and offer the youngster a sense of agency [7].

Distraction strategies such as watching cartoon movies, parents' verbal interactions, the use of party blowers, etc., which are linked to the gate control theory, include shifting the child’s focus from the source of discomfort to something else. They think that diversion can alter how the brain deals with painful signals. During times of distraction, the area of the brain responsible for registering painful experiences receives less blood [8]. When we take a person’s focus away from an unpleasant sensation, such as pain, the activity in the thalamus, insula, and anterior cingulate cortex decreases, and the person perceives less discomfort [9].

For a long time, we have conducted research on the pharmacological methods of using a local anesthetic mixture for venipuncture. They have shown two percent lidocaine in studies to be effective in lowering pain and distress during procedure discomfort, and they advised it for everyday use in pediatric wards with few adverse effects like erythema, edema, pruritis, etc. [10]. It is suggested that local anesthetic mixture cream be used to alleviate the discomfort of venipuncture and Intravenous (IV) insertion. In healthcare systems with limited resources, the cost of a single local anesthetic mixture cream application may be prohibitive. However, healthcare professionals should identify patients at high risk for IV insertion pain and its associated adverse effects.

Healthcare practitioners can use distraction as a non-pharmacologic strategy to control and lessen anxiety, notably for painful pediatric treatments [11,12]. Many studies have shown that both passive and active forms of distraction such as reading, playing video games, or watching television reduce feelings of discomfort and anxiety. Virtual reality may provide even more distraction because it puts the patient in a different world and uses more than one sense [13].

Using distraction techniques involves directing the child's attention away from their pain experience and toward the distraction, which is related to the gate control theory [14-16]. Therefore, this study aims to compare the efficacy of pain reduction by using animation distraction versus two percent lignocaine with a control group undergoing intravenous cannulation among children in a pediatric ward, Intensive Care Unit (ICU), or emergency department and to study the clinico-social factors influencing pain reduction among them, which further helps in early cannulation and easy drug administration and management of the child.

## Materials and methods

Study design and setting

Children between the ages of six and twelve who were admitted to the paediatric ward, intensive care unit (ICU), or emergency room under the care of the Department of Pediatrics at Malankara Orthodox Syrian Church (MOSC) Medical Mission Hospital in Kolenchery, Kerala, India from June 2021 to June 2022 were included in an open-label randomized controlled trial study. This tertiary care facility has 100 beds in the general ward and 15 beds in the intensive care unit, admitting between 60 and 90 patients per month.

Study subjects

We assigned children to either two percent lignocaine application, animation distraction, or control during intravenous cannulation (divided into three groups with n = 20 in each group).

Sampling procedure

Convenient sampling with randomization for the allocation of study groups and control groups using the block randomization technique. We began with ten blocks and six sequences, and with the assistance of an online random block randomization generator, we were able to construct a variety of different sequences. Children were divided into intervention groups using the generated sequences. 

Inclusion criteria

We included in the study those children aged 6-12 years whose parents gave their consent, whose first IV cannulation happened during the present admission, and for whom we successfully placed the cannula on the first or second try.

Exclusion criteria

We excluded children who presented with severe illness, had neurodevelopmental impairment or cerebral palsy, consumed paracetamol or any anti-inflammatory medication within the previous two hours, or had a history of an allergic reaction to local anesthetics, congenital or idiopathic methemoglobinemia, glucose-6-phosphate deficiency, or severe hepatic disease.

Sample size

Based on the results of the randomized controlled study by Balan et al. in the inpatient department of a tertiary care centre in Mumbai [17], we calculated the sample size by using the formula,

$n=(2\rho ^2 (Z_{(1-\frac{\alpha }{2})}+Z_{1-\beta } )^2*[1+(m-1)\rho ]/(md)^2$

m = number of repetitions, ρ = Intraclass correlation coefficient (0.3), d = clinically significant difference σ = Pooled standard deviation

Hence, σ = 2

m=2, Z_1-α/2_*m=1.96, Z_1-β_=0.89, μd^2^=1

Therefore, n=20 in each group

We calculated the sample size to be twenty in each group.

Ethical considerations

Before beginning the study, we received approval from the Institutional Ethical Committee and Institutional Research Board at the Department of Pediatrics, MOSC Medical Mission Hospital, Kolenchery in Kerala (Approval Number: MOSC/IEC/544/2021). The parents of the participants gave their informed, written consent. We registered this study under the Clinical Trials Registry-India (CTRI), and the CTRI Registration No. is REF 2021/06/044578.

Study tools

A proforma was used to collect clinico- social data about children. The visual analogue scale was used to measure the subjective pain intensity of the children during IV cannulation. The visual analogue scale is a sheet of paper with a 100-mm horizontal line at one end representing no pain and the other end representing extreme pain.

Methods of data collection

We got written informed consent from parents and then we randomized children according to a computer-generated permuted block randomization into either of the three groups. The investigator assigned groups by using numbered, opaque, sealed envelopes. Before entering the procedure room, the envelopes were opened. We explained the VAS score to the child prior to the procedure. We took the VAS recording at zero seconds, one minute, and five minutes.

Distraction group

We took the children to the procedure room and seated them with their arms placed on the table. We avoided using pre-treatment with paracetamol or an anti-inflammatory in children for pain. The intervention used for the distraction group was a nature animation with both entertaining and educational value that lasted five minutes and it was played on a laptop. We started showing the animation from a laptop screen at a comfortable distance one minute before the needle prick. We took the VAS recording at zero seconds, one minute, and five minutes.

Local anesthetic group

On the children, we applied the topical anesthetic agent, lignocaine, at two percent as a thick layer of gel or cream, then secured it with an occlusive dressing to facilitate absorption by the skin for 45 minutes prior to cannulation. Parents should watch their children to make sure they do not rub their eyes. At zero seconds, one minute, and five minutes, we recorded the VAS.

Control group

We seated the children with their arms resting on the table in the procedure room. To the extent possible, we held off on giving children painkillers like paracetamol or ibuprofen before their procedure. We did not deal with the use of topical local anesthetic. At zero seconds, one minute, and five minutes, we took the VAS recording.

Statistical methods

We entered the collected data into Microsoft Excel (Microsoft Corporation, Redmond, USA), cleaned it, and analyzed it using SPSS version 21 (IBM Corp., Armonk, NY). Means and standard deviations are used to depict numerical variables. Frequency and percentage displays are used to depict categorical variables. We conducted an ANOVA test to compare the means of numerical variables across the lignocaine, animation distraction, and control groups. When comparing two categorical variables, we employed either the Fisher’s exact or chi-square test to establish statistical significance. We employed an independent t-test to determine statistical significance when comparing a numeric variable to categories with two study outcomes. We considered p-values less than 0.05 to be statistically significant.

## Results

The lignocaine group had an 8.35 mean age (years) compared to 8.1 for controls and 7.95 for distraction, although the difference was not statistically significant (p > 0.05). The lignocaine group had 35% male subjects, 60% in the distraction group, and 35% in the control group. It was not statistically significant. More children with graduate mothers were present in the control group (30%) and the distraction group (30%), whereas 25% of the participants' mothers in the lignocaine group were middle schoolers. The distribution of moms with different educational levels was not statistically significant (p > 0.05). Forty percent of the lignocaine group, 35% of the distraction group, and 15% of the control group had a history of** **cannulation, however, the difference was not statistically significant (p > 0.05). We cannulated 45 percent of children in the lignocaine group with a 22-gauge needle, compared to 35% in the distraction group, 35% in the control group, and the rest with a 24-gauge needle. The difference was not statistically significant (p > 0.05). Twenty-five percent of the lignocaine group had cephalic cannulation, 40% in the distraction group, and 45% in the control group, while the rest received metacarpal cannulation. It was not statistically significant. We have described the association between the socio-demographic variable and intervention groups among study participants in Table 1.

**Table 1:** Association between the socio-demographic variable and intervention groups among study participants *One-way ANOVA used

Variable		Lignocaine	Distraction	Control	Total	P value
Age		8.35 ± 2.08	7.95 ± 1.70	8.1 ± 1.45	60 (100%)	0.77*
Gender	Male	7 (35%)	12 (60%)	7 (35%)	26 (43.33%)	0.184
	Female	13 (65%)	8 (40%)	13 (65%)	34 (56.66%)	
Mothers Educational Status	Primary School	3 (15%)	6 (30%)	3 (15%)	12 (20%)	0.778
	Middle School	5 (25%)	4 (20%)	4 (20%)	13 (21.66%)	
	High School	5 (25%)	1 (5%)	4 (20%)	10 (16.66%)	
	Graduate	4 (20%)	6 (30%)	6 (30%)	16 (26.66%)	
	Postgraduate	3 (15%)	3 (15%)	3 (15%)	9 (15%)	
Previous history of Cannulation	Yes	8 (40%)	7 (35%)	3 (15%)	18 (30%)	0.189
	No	12 (60%)	13 (65%)	17 (85%)	42 (70%)	
Size of Cannula	22 Gauge	9 (45%)	7 (35%)	7 (35%)	23 (38.33%)	0.755
	24 Gauge	11 (55%)	13 (65%)	13 (65%)	37 (61.66%)	
Site of Cannula	Cephalic	5 (25%)	8 (40%)	9 (45%)	22 (36.66%)	0.394
	Metacarpal	15 (75%)	12 (60%)	11 (55%)	38 (63.33%)	

The distraction group’s mean VAS score at zero minutes was 2.25, which was significantly lower than the lignocaine group’s 5.55 and the control group’s 6.15 (p < 0.05). At one minute, the mean VAS score for the distraction group was 3.65, compared to 5.75 for the lignocaine group and seven for the control group. The difference was statistically significant (p < 0.05) by one-way ANOVA with Bonferroni post hoc test. After five minutes, the distraction group’s mean VAS score was 3.35, which was lower than the lignocaine and the control group's scores of 5.85 and 7.15. (p < 0.05). We have described the association between VAS score and intervention groups among study participants in Table 2.

**Table 2:** Association between the VAS score and intervention groups among study participants by one-way ANOVA test VAS - Visual Analogue Scale

VAS score	Lignocaine (µ±σ)	Distraction (µ±σ)	Control (µ±σ)	P value
Zero min	5.55 ± 1.10	2.25 ± 1.55	6.15 ± 1.57	0.001
One min	5.75 ± 0.91	3.65 ± 1.63	7.00 ± 1.38	0.001
Five min	5.85 ± 1.39	3.35 ± 1.76	7.15 ± 1.53	0.001

The mean VAS score at zero minutes was significantly different between lignocaine and distraction and distraction and control. Lignocaine, distraction, and control had statistically significant mean VAS scores at one minute. The mean VAS score at five minutes differed significantly between lignocaine, distraction, and control. These differences were statistically significant (p < 0.05) by one-way ANOVA with Bonferroni post hoc test. Using a one-way ANOVA test, we describe in Table 3 the association between VAS score and intervention group within study participants.

**Table 3:** Association of VAS scores within intervention groups among study participants by one-way ANOVA test VAS - Visual Analogue Scale

VAS score	Intervention groups	Mean difference	Standard Error	P value
VAS score at zero min	Lignocaine vs Distraction	3.3	0.45	0.001
	Lignocaine vs Control	0.6	0.45	0.562
	Distraction vs Control	3.9	0.45	0.001
VAS score at one min	Lignocaine vs Distraction	2.1	0.42	0.001
	Lignocaine vs Control	1.25	0.42	0.014
	Distraction vs Control	3.35	0.42	0.001
VAS score at five min	Lignocaine vs Distraction	2.5	0.49	0.001
	Lignocaine vs Control	1.3	0.49	0.033
	Distraction vs Control	3.8	0.49	0.001

After lignocaine, the mean pulse rate was 93.1, which was not statistically different from 91.2 before. The mean pulse rate before distraction was 90.45 and after distraction was 95.25, a statistically significant variation. Control groups had a mean pulse rate of 92.55 before intervention and 93.4 after, but the difference was not statistically significant by paired T-test. Before lignocaine, the mean saturation of peripheral oxygen (SpO2) was 97.2; after, it was 97.45. The change was not statistically significant by paired t-test. Before distraction, the mean SpO2 was 88.8, and after distraction, it was 97.25. The difference was not statistically significant by paired t-test. The control groups had a mean SPO2 of 97.35 before and 96.95 after, but the difference was not statistically significant by paired t-test. We have described the changes in pulse rate and SpO2 before and after intervention in Table 4.

**Table 4:** Changes in pulse rate and SpO2 before and after intervention by paired t-test Spo2 - saturation of peripheral oxygen

Variable	Intervention groups	Before intervention	After intervention	Mean difference	P value
Pulse Rate	Lignocaine	91.20 ± 4.86	93.10 ± 4.80	1.9	0.252
	Distraction	90.45 ± 6.24	95.25 ± 3.71	4.8	0.012
	Control	92.55 ± 5.34	93.40 ± 5.28	0.85	0.662
SpO_2_	Lignocaine	97.20 ± 1.47	97.45 ± 1.05	0.25	0.489
	Distraction	88.80 ± 27.32	97.25 ± 1.55	8.45	0.19
	Control	97.35 ± 1.53	96.95 ± 1.64	0.4	0.423

Each age group had similar VAS scores. Male and female VAS scores at zero, one, and five minutes differed significantly. VAS scores were unaffected by the mother’s education. Children who had a history of** **cannulation had no significant difference in VAS scores at zero, one, and five minutes, but those who had not had it had a significant difference from zero to five minutes. Children using 22-gauge cannulas had a substantial VAS score increase from zero to one minute and a decrease at five minutes. The VAS score of children receiving 24-gauge cannulas increased significantly from zero to one to five minutes. The VAS score of children with cephalic and metacarpal cannulation increased from zero to one minute and reduced at five minutes, which was significant. Using a one-way ANOVA test, the association between VAS score and socio-demographic characteristics within each group is described in Table 5.

**Table 5:** Association between VAS score and socio-demographic characteristics within the groups by one-way ANOVA test. VAS - Visual Analogue Scale

Variable		VAS score			P value within group
		zero min	one min	five min	
Age	5	2 (±0)	4 (±0)	5 (±0)	--
	6	4.3 (±2.3)	6.1 (±1)	5.5 (±2.2)	0.052
	7	5.5 (±2.4)	5.6 (±2.3)	5.9 (±2.6)	0.512
	8	4.7 (±2.5)	5.3 (±2.4)	5.3 (±2.8)	0.163
	9	3.7 (±2)	4.6 (±1)	4.1 (±0.9)	0.476
	10	4.2 (±1.6)	5.5 (±1.9)	5.5 (±1.9)	0.223
	11	5 (±2.4)	5.8 (±2.3)	5.8 (±1.9)	0.274
	12	5 (±1.4)	5 (±0)	6.5 (±0.7)	--
Gender	Male	4.4 (±2.3)	5 (±2.1)	5 (±2.4)	0.017
	Female	4.8 (±2.2)	5.8 (±1.8)	5.8 (±2)	0.004
Mother's educational status	Primary School	4.1 (±2.4)	4.8 (±2)	4.6 (±2.5)	0.089
	Middle School	4.5 (±2.1)	5 (±1.8)	5.2 (±2.2)	0.287
	High School	5.7 (±1.7)	6.8 (±1.3)	6.3 (±1.9)	0.196
	Graduate	4.8 (±2.4)	5.6 (±2.1)	5.4 (±2.4)	0.143
	Postgraduate	4.2 (±2.4)	5.4 (±1.9)	6.1 (±1.6)	0.082
Previous history of annulation	Yes	4.4 (±2.3)	5.1 (±2)	5.2 (±2.3)	0.094
	No	4.7 (±2.2)	5.6 (±1.9)	5.5 (±2.2)	0.001
Size of Cannula	22 Gauge	4.3 (±2.5)	5.4 (±1.6)	4.8 (±2.2)	0.03
	24 Gauge	4.8 (±2.1)	5.5 (±2.1)	5.8 (±2.1)	0.001
Site of Cannula	Cephalic	4.1 (±2.8)	5.2 (±2.2)	5.4 (±2.4)	0.011
	Metacarpal	4.9 (±1.8)	5.6 (±1.8)	5.5 (±2.1)	0.004

Children of different ages had similar VAS ratings. VAS scores at zero, one, and five minutes were similar for boys and girls. VAS scores were unaffected by the mother’s education. Both groups of non-cannulated patients had similar VAS scores. The VAS values showed no significant cannula size difference. VAS values were similar among cannula locations. In Table 6, we describe the association between VAS score and socio-demographic characteristics across groups using the one-way ANOVA test.

**Table 6:** Association between VAS score and socio-demographic characteristics between the groups by one-way ANOVA test VAS - Visual Analogue Scale

Variable	VAS score			ANOVA
	0 min	1 min	5 min	
Between Age	0.626	0.823	0.816	P value
Between gender	0.494	0.132	0.136	
Between Mother's educational status	0.486	0.115	0.379	
Previous history of cannulation	0.643	0.281	0.606	
Between the size of cannula	0.411	0.813	0.085	
Between site of cannula	0.228	0.386	0.82	

## Discussion

Baseline characteristics influencing the study outcomes

In this study, age had a poor correlation with the VAS score at zero, one, and five minutes after venipuncture. There was no significant difference in VAS scores between the ages of the children and within each age group. Giorgio Cozzi et al. found in their study that adolescent patients had high levels of pre-procedural distress and comparable degrees of pain and anguish to younger patients [18].

In this study, the VAS scores within males and within females at zero, one, and five minutes had statistically significant differences. There was no significant difference between male and female children in VAS scores at zero, one, and five minutes. In the study by Jagadamba et al. in India, published in 2010, females reported severe pain perception, whereas males reported withdrawal [19].

We compared the educational status of mothers in this study because there was a possibility of pre-canulation priming by mothers of their children, which could affect the VAS score. In the study by Kleiber et al., in the USA in 2001, they employed parental distraction coaching during IV cannula insertion [20].

Basavana Gouda Goudra et al. concluded in their study that the antecubital fossa should be the cannulation site of choice over the dorsum of the hand, considering the VAS pain scores between the groups [21]. In this study, the site of the cannula did not significantly influence the VAS scores between the groups. Although we have noted increased heart rate in studies by Bartfeild et al. [22] and Kartufan [23], we have not found it to reduce pain significantly. In this study, we observed a rise in pulse rate in all three groups, but the difference in pulse rate before and after cannulation was statistically significant only in the distraction group.

VAS score

In this study, the mean VAS score in the distraction group was lower than the mean in the lignocaine group and the control group at zero, one, and five minutes. The difference in mean VAS score at zero, one, and five minutes was statistically significant in lignocaine vs. distraction, lignocaine vs. control, and distraction vs. control groups.

Similar to our study results (superiority of the animation distraction over the control group), JA Vessey et al. compared the animation distraction for reduction of pain relief and noticed that, compared to the control group, the experimental group reported less pain and showed less behavioral discomfort [9].

Lobo and Umarani noticed that cartoon distraction helped children who were having venipuncture procedures feel less pain since it decreased their pain perception score on the Wong-Baker FACES Pain scale [24].

Shivcharan Singh Gandhar et al. noticed that cartoon distraction helped children who were having venipuncture procedures feel less pain since it decreased their pain perception score on the observational pain scale [25].

Thakur P et al. observed that a cartoon animation movie significantly decreased the level of pain during the intravenous cannulation procedure among the children in the experimental group as compared to the control group since it decreased their pain perception score on the observational pain scale [26].

Similar to our study results (the superiority of the animation distraction over lignocaine), Cohen et al. observed among the 39 young children undergoing IV cannulation that cartoon distraction is more effective in reducing distress in children undergoing procedures under local anesthesia [27].

Fatma Güdücü Tüfekci et al., using different distraction techniques, assessed pain using the Wong-Baker FACES Pain Rating Scale and the Visual Analogue Scale. They found that the diversion created by a kaleidoscope significantly decreased the pain associated with venipuncture in healthy school children [28].

A study by Biji S et al. compared the animation distraction with the control group, in which they observed that the intervention group's lower FACES pain score (at zero, one, and three minutes) demonstrates how successfully animation distraction reduces pain when cannulating [29]. This shows the reliability of the study results of animation distraction for pain reduction.

Limitations

We could not do blinding in this study because the interventions varied from the modes. The study may have subject and interviewer bias. The sample size was less for evaluating the connections between individual characteristics and study variables. Animation affects children differently, depending on their geography, culture, and hobbies. Thus, these interventions are questionable. We did a hospital-based study in a tertiary care setting; hence it had superior study settings and expertise than other studies.

## Conclusions

Based on the findings, we conclude that age, gender, history of cannulation, site, and size of the cannula were not significantly different between the groups. We significantly related the education of the mother to the VAS scores but did not display any linear relationship. The mean VAS score for pain at zero minutes, one minute, and five minutes was significantly lower in the distraction group compared to the lignocaine and control groups. Therefore, animation distraction is preferable to lignocaine to alleviate pain in children requiring IV cannulation for a variety of invasive procedures, as it is effective and has no adverse effects. Further research with a larger sample size and different distractions, conducted in other healthcare settings, such as secondary care, will show the true efficacy and replicability of the study results.
